# Efficacy and safety of Chinese herbal medicine to prevent and treat COVID-19 household close contacts in Hong Kong: an open-label, randomized controlled trial

**DOI:** 10.3389/fimmu.2024.1359331

**Published:** 2024-05-10

**Authors:** Peipei Du, Wai Ching Lam, Choryin Leung, Huijuan Li, Zipan Lyu, Chun Sum Yuen, Chun Hoi Cheung, Tsz Fung Lam, Zhaoxiang Bian, Linda Zhong

**Affiliations:** ^1^ School of Chinese Medicine, Hong Kong Baptist University, Hong Kong, Hong Kong SAR, China; ^2^ Biomedical Sciences and Chinese Medicine, School of Biological Sciences, Nanyang Technological University, Singapore, Singapore

**Keywords:** Chinese herbal medicine, COVID-19, prevention, treatment, randomized controlled trial

## Abstract

**Objectives:**

To evaluate the efficacy and safety of CHM in the prevention of COVID-19 infection and treatment for COVID-19 related symptoms.

**Design:**

Prospective open-label randomized controlled trial.

**Setting:**

Participants’ home in Hong Kong.

**Participants:**

Participants who had household close contact with COVID-19-infected family members.

**Interventions:**

Close contacts were stratified into 4 groups (cohort A, B, C, D) based on symptoms and infection status and were randomized in 4:1 ratio to receive CHM granules (9g/sachet, two times daily) or blank control for 7 days with 2 weeks of follow-up.

**Main outcome measures:**

The primary outcome measure was the rate of positive nucleic acid tests. Secondary outcomes were the proportion of developed COVID-19 related symptoms and adverse events during the whole 3-week study period. Subgroup analysis was used to evaluate demographic factors associated with positive infection rates.

**Results:**

A total of 2163 contacts were enrolled and randomly assigned to the CHM group (1720 contacts) and blank control (443 contacts) group. During the 21 days, the rate of PCR-positive cases in cohort A was markedly lower in the CHM group (3.6%) compared to the control group (7.0%) (*P*=0.036). Overall, the rate of infection in the CHM group was significantly lower than that in the control group (10.69% *vs*. 6.03%; RR 0.56, 95% CI 0.39-0.82) after 7-day treatment. No serious adverse events were reported during the medication period.

**Conclusion:**

The preliminary findings indicate that CHM may be effective and safe in preventing COVID-19. Future double-blind, randomized controlled trials and long-term follow-up are needed to fully evaluate the efficacy of CHM in a larger contact population.

**Clinical trial registration:**

ClinicalTrials.gov, identifier NCT05269511

## Introduction

Several notable variants of severe acute respiratory syndrome coronavirus 2 (SARS-CoV-2), including Alpha, Beta, and others, have caused significant outbreaks of infection since the emergence of the ancestral strain [SARS-CoV-2 wild type (WT)] in late 2019 (1). The Omicron variant, first identified in South Africa and Botswana in November 2021, quickly became dominant across the world, causing global panic and concern due to its increased contagious and enhanced vaccine-escape mutations (2, 3).

The Omicron variant (BA.1) was first detected in Hong Kong among two fully vaccinated travelers in a hotel quarantine in November 2021 (4). Subsequently, a small community outbreak occurred in early January 2022, associated with 2 aircrew members infected overseas (5). Local cases of Omicron BA.2 were reported in another quarantine hotel in mid-January 2022 (6). Ultimately, a large fifth wave dominated by BA.2 peaked in early March 2022 in Hong Kong (7). Despite a series of intensive public health and social measures (PHSMs) that included quarantine and isolation, limitations on social gatherings, and restrict travel and border controls, these measures failed to avert the massive epidemic as the fifth wave began (8). The exponential increase in confirmed Coronavirus disease 2019 (COVID-19) cases overwhelmed the healthcare systems (9). The “StayHomeSafe” scheme was launched on 8 February 2022 as a viable cost-effective alternative. Under this scheme, close contacts and COVID-19 confirmed cases who had to undergo 14-day or 4-day quarantine shifted from staying at quarantine facilities to home quarantine (10). This change highlighted the critical issues of preventing COVID-19 infections among household close contacts and providing early treatment for potential COVID-19 symptoms.

Vaccination is currently the most recommended way to prevent COVID-19. Hong Kong’s COVID-19 vaccination program was launched in February 2021 (11). As of 20 April 2022, 80% and 41% of the population had been immunized with the 2- and 3- doses of vaccine, respectively (12). However, the presence of multiple SARS-CoV-2 variants may result in lower vaccine efficacy/effectiveness (VE) (13). A previous study showed that during the BA.2 periods, VE declined to 24% after ≥150 days following the second vaccine dose and to 52% after ≥120 days following the third dose (14). Furthermore, the induced immunity by the third BNT162b2 vaccine dose was found to be transient in elderly individuals (15). As we know, early cases isolated under home quarantine mainly rely on general over-the-counter medications to alleviate of COVID-19 symptoms. However, these drugs often fail to cover the majority of the discomforts and may have additional side effects (16–18). Accumulating evidence has shown that early intervention with Traditional Chinese medicine (TCM) in COVID-19 patients is important to improve the cure rate, shortening the disease course, delaying disease progression, reducing the mortality rate (19, 20). The pharmacological mechanisms of TCM against COVID-19 were mainly attributed to the down-regulated virus activity, inhibited cytokine storm, and enhanced immune function (21). Several Chinese herbal compounds have been shown to inhibit the enzymatic activity of SARS-CoV-2 3CLpro, which is critical for virus replication and activity, while others may prevent SARS-CoV-2 infection by targeting the host receptor ACE2, the cellular entry point for SARS-CoV-2 (22, 23). However, the demonstrated efficacy of TCM for COVID-19 has mostly focused on confirmed patients, lacking sufficient supporting evidence regarding its efficacy in preventing infections among close contacts.

In this study, we utilized a Chinese herbal medicine (CHM) formula consisting of 10 herbs to prevent the infection and progression of mild cases. This formula was derived by modifying 2 classical formulae reported to be efficacious in alleviating COVID-19 symptoms (23–25). The purpose of this study was to evaluate the efficacy and safety of this Chinese herbal medicine in the prevention of COVID-19 and treatment for COVID-19 related symptoms to provide real-world evidence for clinical prophylaxis.

## Methods

### Study design

This is a randomized, blank-controlled study in adults with household contact exposure to individuals with SARS-CoV-2 infection. This study received approved by the Hong Kong Baptist University Research Ethics Committee (REC) in compliance with scientific content and applicable research and human subjects’ regulations (Ethics approval number: REC/21-22/0349). All subjects were household contacts with close exposure to household member known to be infected with SARS-CoV-2. After the subjects provided the informed consent, they were assessed online for study eligibility by a Chinese Medicine Practitioner (CMP). After the screening, eligible subjects were divided into 4 cohorts based on the results of the PCR during screening and the presence or absence of mild symptoms in the patients. Then, subjects were randomly assigned to either the control group or the treatment group. The treatment period lasted 7 days, as Omicron variant is characteristically most infectious within one week of exposure (26), and the follow-up period was 14 days. Subjects were required to record their symptoms/signs throughout the whole study. The investigators collected records by telephone and mail at each follow-up time point.

### Participants

Recruitment took place between Mar 2022 to June 2022 through newspaper advertisements and social network platforms. Individuals interested in the trial could contact the investigators by telephone, e-mail, or online registration. Volunteers were considered for inclusion if they met the following criteria: (1) exposure to cohabitants with SARS-CoV-2 infection; (2) aged ≥16; (3) were general healthy or had a chronic, stable medical condition; (4) signed a written informed consent form voluntarily; (5) able to follow written and oral instructions in Chinese. Participants with any of the following conditions were excluded: (1) Moderate to severe symptomatic SARS-CoV-2 infection with hospitalization; (2) Allergic history to Chinese herbals or a known allergy to the ingredients of the study CHM; (3) Pregnancy, breastfeeding, or plan to become pregnant within the study time frame; (4) Vulnerable adults (i.e., mentally or physically disabled to take care of himself/herself); (5) Any physical examination findings, and/or history of any illness, or concomitant medications that, in the opinion of the study investigator, might not be suitable to participate in the study.

CMPs prescribed the CHM to eligible subjects after the remote visit and randomization. One-week dosage of CHM was mailed to the subjects within a day. Allocation and recipient records, including quantity, code number, and date, were documented and signed off by authorized research personnel. Only the authorized researchers in this study could distribute the products to the participants as per instructions from the PI, after confirming the randomization code. It was the researchers’ responsibility to guide the participants on the administration method and precautions. The School of Chinese Medicine’s pharmacy was responsible for allocating and managing the investigation CHM.

### Sample size calculation

The calculation of sample size was based on a previously study that the rate of positive nucleic acid detection in close contacts of COVID-19 patients was approximately 7.6% (27). Researchers assumed that the positive rate would be 3.5% after the CHM intervention. The ratio between the groups was set at 4:1. Therefore, a sample size of 1728 participants in the CHM treatment group and 432 participants in blank control group was needed with 80% power, assuming a significance level of 0.05 and 15% attrition rate. We used the calculation tool available at http://powerandsamplesize.com/Calculators/.

### Randomization and blinding

Stratified randomization was carried out in a 4:1 ratio, assigning subjects to either the CHM treatment group or the blank control group. The random grouping program was set up by statistical professionals using random sequences generated by R (version 4.1.0). Randomization was performed based on symptoms and infection as follows: (1) Cohort A: adults who tested SARS-CoV-2 PCR negative and were asymptomatic at baseline; (2) Cohort B: adults who tested SARS-CoV-2 PCR negative but displayed COVID-19 symptoms at baseline; (3) Cohort C: adults who tested SARS-CoV-2 PCR positive but were asymptomatic at baseline; (4) Cohort D: adults who tested SARS-CoV-2 PCR positive and displayed COVID-19 symptom at baseline. Participants and investigators were not blinded to the group assignment and treatment allocation.

### Intervention

The intervention of CHM was in the form of granules (9 g/sachet), which were comprised of ten medicinal herbs: *Pseudostellariae Radix, Lonicerae Japonicae Flos, Forsythiae Fructus, Isatidis Radix, Mori Cortex, Schizonepetae Herba, Glycyrrhizae Radix et Rhizoma, Poria, Atractylodis Macrocephalae Rhizoma, Platycodonis Radix*. Detailed information regarding the name and dosages of each ingredient can be found in **Table 1**. None of the ten herbs were endangered species or grown in a threatened habitat. The study’s CHM were stored at the pharmacy of the School of Chinese Medicine at HKBU at room temperature until use. During the 1-week intervention, participants were required to consume a total of 18g of granules per day, 1 hour after breakfast and 1 hour after dinner, by dissolving 1 sachet of 9g of granules in 150 ml of hot water and drinking. All the granules were provided by a GCP manufacturers and fulfilled the quality control standards set by the Hong Kong SAR government. Participants in the control group were under medical observation.

**Table 1:** The composition of Chinese Herbal Medicine.

Latin name	Chinese name (Pinyin)	g/day
*Pseudostellariae Radix*	Taizishen	10
*Lonicerae Japonicae Flos*	Jinyinhua	10
*Forsythiae Fructus*	Lianqiao	10
*Isatidis Radix*	Banlangen	10
*Mori Cortex*	Sangbaipi	10
*Schizonepetae Herba*	Jingjie	5
*Glycyrrhizae Radix et Rhizoma*	Gancao	10
*Poria*	Fuling	10
*Atractylodis Macrocephalae Rhizoma*	Baizhu	5
*Platycodonis Radix*	Jiegeng	10

In order to maximize patients’ compliance, we will firstly run a thorough consent process for all participants by explaining the details of the study schedule, potential side effects of treatment, insurance, and the responsibilities that need to be undertaken by subjects. Secondly, support and reassurance are provided during the whole study. Thirdly, we will carefully scrutinize the subjects (during the run-in period) to exclude ineligible and potentially low compliance individuals before randomization. Fourthly, a direct telephone hotline and email account will be established in order to optimize active communication with patients and to respond to enquiries. If any patient expressed thoughts of withdrawing or dropping out, we would try to help that individual determine the reason, and attempt to resolve any issues, in order to keep the patient in the study. In order to monitor the reported compliance of the patients, the patients will be required to return the remaining medications at treatment visits. Investigators and CMPs will take a record of each patient. 80% of the consumption will be considered as good compliance.

### Outcomes

The primary outcome was the proportion of contacts who tested positive SARS-CoV-2 infection after 7 days of treatment, with details of the testing method recorded (for example, PCR test or SARS-CoV-2 antigen test through nasal or saliva samples). The secondary outcome was the proportion of subjects who developed symptoms (including fever, cough, headache, or fatigue) after the 7-day treatment period. The main analysis of safety outcomes was based on adverse events during the medication.

### Statistical analysis

Continuous variables are presented as mean ± standard deviation (SD) or median with interquartile range [median (IQR)], as appropriate. Categorical variables were described using frequencies and percentages. General demographic characteristics were analyzed in different groups by the two-sample Student’s t-test or Chi-square test. Analyses used an intention-to-treat (ITT) population approach comprising all randomized participants. The last PCR test or SARS-CoV-2 antigen test result served as projected end points for those who withdrew prior to the posttreatment assessment (Last Observation Carried Forward method). Subgroup analysis was conducted by gender (male and female), age (<46 years, 46-60 years, 61-75years, 75 years), BMI (<18.5 kg/m2, 18.5-23.9 kg/m2, 24-28 kg/m2, ≥28 kg/m2), Number of vaccines (0, 1, 2, 3), complication (no, yes) and clinical symptom of COVID-19 (no, yes) to further elucidate the effect of contacts characteristics on the positive rate of nucleic acid test in different subgroups of the population. Multivariate logistic regression was used to examine the relationship between contacts characteristics and PCR positive in CHM group. Odds Ratio (OR) and 95% confidence interval (95% CI) were calculated for the variables. All the statistical analyses were processed using the R software (version 4.1.0). A two-sided *P*-value <0.05 was considered statistically significant.

## Results

**Figure 1:**
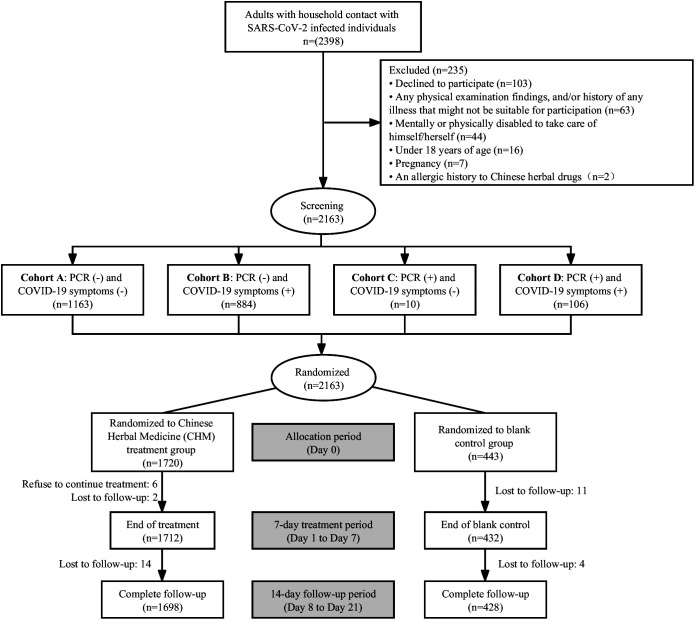
Study flowchart.

A total of 2398 participants who had contacted with SARS-CoV-2 infected individuals were assessed for trial eligibility. Out of these, 2163 contacts signed informed consent and were enrolled and randomly assigned to either the CHM group (1720 contacts) or the blank control (443 contacts) group (**Figure 1**). All the contacts were assessed at baseline according to their COVID-19 PCR test results and symptoms/signs. Of the 2047 (94.6%) contacts who tested negative for PCR, of which 1163 (56.8%) were asymptomatic and 884 (43.2%) were symptomatic. On the other hand, 116 (5.4%) contacts tested positive for PCR, of whom 106 (91.4%) were symptomatic and only 10 contacts were asymptomatic. Each contact received either CHM treatment or blank control for one week, with a 14-day follow-up. There were 37 drop-out cases in this study, with 22 (59.5%) in the CHM group and 15 (40.5%) in the blank control group, and a total of 2126 contacts completed this study. **Figure 1** shows the flow diagram for this randomized controlled trial.

### Contacts characteristics

The baseline characteristics of contacts in both the control and CHM groups are shown in **Table 2**. The majority of participants were females, accounting for 1466 (67.8%) of the total. The proportion of females in the CHM group was slightly higher at 70.0% compared to the control group’s 67.2%. The mean age of all contacts was 59.08 years, ranging from 18 to 96 years. The mean age was 59.08 years in the control group and 59.12 years in the CHM group. Compared to the control group, the CHM group exhibited a similar mean value for BMI and Body temperature. A total of 104 (4.8%) contacts had a baseline body temperature above 37°C, with 27 (6.1%) in the control group and 77 (4.5%) in the CHM group. The mean number of COVID-19 vaccines administered to all contacts is 2.55. 1128 (52.1%) contacts had one or more complications, and 990 (45.8%) contacts reported clinical symptoms of COVID-19 at baseline. No statistically significant differences were found in baseline variables between these two groups (**Table 2**).

**Table 2:** Baseline characteristics of contacts in CHM and placebo groups.

Characteristics	Control group n=443	CHM group n=1720	*P* value
**Gender, n (%)**			0.292
**Male**	133 (30.0)	564 (32.8)	
**Female**	310 (70.0)	1156 (67.2)	
**Age, mean (SD), y**	59.12 (16.64)	59.08 (16.89)	0.964
**Age-group, n (%)**			0.330
**<46**	102 (23.0)	423 (24.6)	
**46-60**	100 (22.6)	323 (18.8)	
**61-75**	179 (40.4)	736 (42.8)	
**>75**	62 (14.0)	238 (13.8)	
**BMI, mean (SD), kg/m^2^**	23.52 (3.55)	23.41 (3.36)	0.533
**BMI-group, n (%)**			0.844
**<18.5**	256 (57.8)	996 (57.9)	
**18.5-23.9**	15 (3.4)	73 (4.2)	
**24-28**	134 (30.2)	500 (29.1)	
**≥28**	38 (8.6)	151 (8.8)	
**Body temperature, mean (SD), °C**	36.48 (0.46)	36.46 (0.59)	0.662
**Body temperature >37°C, n (%)**			0.195
**No**	416 (93.9)	1643 (95.5)	
**Yes**	27 (6.1)	77 (4.5)	
**Number of vaccines, n (%)**	2.59 (0.75)	2.54 (0.82)	0.238
**First dose, n (%)**			0.063
**BNT162b2**	204 (48.2)	681 (42.0)	
**CoronaVac**	219 (51.8)	940 (58.0)	
**Beijing biology**	0	1 (0.1)	
**Second dose, n (%)**			0.057
**BNT162b2**	202 (49.1)	663 (42.7)	
**CoronaVac**	209 (50.9)	889 (57.2)	
**Chengdu biology**	0	1 (0.1)	
**Third dose, n (%)**			0.074
**BNT162b2**	160 (51.0)	539 (45.1)	
**CoronaVac**	154 (49.0)	656 (54.9)	
**Complication, n (%)**			0.792
**No**	209 (47.2)	826 (48.0)	
**Yes**	234 (52.8)	894 (52.0)	
**Hypertension, n (%)**	96 (21.7)	365 (21.2)	0.888
**Hypercholesterolemia, n (%)**	75 (16.9)	253 (14.7)	0.277
**Diabetes, n (%)**	56 (12.6)	187 (10.9)	0.334
**Cardiopathy, n (%)**	21 (4.7)	55 (3.2)	0.153
**Clinical symptoms of COVID-19, n (%)**			0.251
**No**	229 (51.7)	944 (54.9)	
**Yes**	214 (48.3)	776 (45.1)	

CHM, Chinese Herbal Medicine; N, number; SD, standard deviation; BMI, Body Mass Index.

### Primary outcomes

The number of SARS-CoV-2 PCR positive cases in both the CHM and control groups in cohorts A, B, C, and D during the treatment and follow-up periods are shown in **Table 3**. In cohort A, the rate of SARS-CoV-2 PCR positive cases in the CHM group, totaling 34 (3.6%), was significantly lower than in the control group, which had 16 (7.0%) cases (*P*=0.036), throughout the 7-day treatment period. Contacts in cohort B were SARS-CoV-2 PCR negative at baseline but had COVID-19 symptoms, with a higher incidence of PCR positive case numbers later. In cohort B, there were 64 (9.3%) SARS-CoV-2 PCR positive cases in the CHM group, whereas the control group had 29 (14.9%) (*P*=0.032). Contacts in group C, who were SARS-CoV-2 PCR positive and asymptomatic at baseline, all turned negative after 7 days of CHM treatment. A total of 116 (5.4%) contacts who were SARS-CoV-2 PCR positive and symptomatic for COVID-19 at baseline. All contacts were PCR negative after 21 days.

**Table 3:** Comparison of the number of SARS-CoV-2 PCR positive cases in the CHM and control groups among the four cohorts.

Groups		SARS-CoV-2 PCR positive contacts, n (%)					
		Baseline	Treatment period		Follow-up period	Overall^a^	*P*-value
		0 day	4 days	7 days	21 days		
**Cohort A**	CHM group (n=936)	0	13	21	0	34 (3.6)	0.036*
	Control group (n=227)	0	7	9	0	16 (7.0)
**Cohort B**	CHM group (n=690)	0	33	31	0	64 (9.3)	0.032*
	Control group (n=194)	0	21	8	0	29 (14.9)
**Cohort C**	CHM group (n=8)	8	1	0	0	1 (12.5)^a^	–
	Control group (n=2)	2	0	0	0	0^a^
**Cohort D**	CHM group (n=86)	86	35	12	0	47 (54.7)^a^	0.158
	Control group (n=20)	20	11	4	0	15 (75.0)^a^
**Overall**		116 (5.4)	121 (5.6)	108 (5.0)	0	229 (10.6)^a^	/

CHM, Chinese Herbal Medicine; PCR, Polymerase Chain Reaction.

*Significant at 0.05; ^a^The total number of SARA-CoV-2 PCR positive cases during treatment and follow-up.

### Secondary outcomes

At baseline, a total of 990 (45.8%) contacts exhibited one or more clinical symptoms of COVID-19 (**Table 4**). In cohort A, the CHM group demonstrated a significantly lower rate of positive clinical symptoms of COVID-19 cases compared to the control group, with 86 (9.2%) versus 33 (14.5%) cases (*P*=0.024) after 4-day treatment. Correspondingly, we observed a relatively better treatment effect in the CHM group compared with control group after 7 days and 21-day follow-up. Similar treatment effects between CHM and the control group were observed for cohort B (33.9% [234/690] *vs*. 42.3% [82/194], *P*=0.039) and D (40.7% [35/86] *vs*. 70.0% [14/20], *P*=0.034) after 4 days. However, for all contacts who exhibited clinical symptoms of COVID-19 at baseline in cohort B and D, the CHM group no longer showed a significant decline in the rate of clinical symptoms positive cases after 7-day treatment or during the follow-up compared to the control group. 103 (4.8%) contacts still reported clinical symptoms of COVID-19 after 21 days.

**Table 4:** Comparison of the number of contacts with positive clinical symptoms of COVID-19 in the CHM and control groups among the four cohorts.

Groups		Contacts with positive clinical symptoms of COVID-19, n (%)			
		Baseline	Treatment period		Follow-up period
		0 day	4 days	7 days	21 days
**Cohort A**	CHM group (n=936)	0	86 (9.2)	123 (13.1)	12 (1.3)
	Control group (n=227)	0	33 (14.5)	102 (44.9)	11 (4.8)
	Overall	0	119 (10.2)	225 (19.3)	23 (2.0)
	*P*-value	/	0.024*	<0.001*	0.001*
**Cohort B**	CHM group (n=690)	690 (100.0)	234 (33.9)	171 (24.8)	54 (7.8)
	Control group (n=194)	194 (100.0)	82 (42.3)	57 (29.4)	21 (10.8)
	Overall	884 (100.0)	316 (35.7)	228 (25.8)	75 (8.5)
	*P*-value	/	0.039*	0.230	0.239
**Cohort C**	CHM group (n=8)	0	3 (37.5)	2 (25.0)	0
	Control group (n=2)	0	1 (50.0)	1 (50.0)	0
	Overall	0	4 (40.0)	3 (30.0)	0
	*P*-value	/	1.000	1.000	–
**Cohort D**	CHM group (n=86)	86 (100.0)	35 (40.7)	26 (30.2)	3 (3.5)
	Control group (n=20)	20 (100.0)	14 (70.0)	9 (45.0)	2 (10.0)
	Overall	106 (100.0)	49 (46.2)	35 (33.0)	5 (4.7)
	*P*-value	/	0.034*	0.317	0.237
**Overall**		990 (45.8)	488 (22.6)	491 (22.7)	103 (4.8)

CHM, Chinese Herbal Medicine.

**Figure 2:**
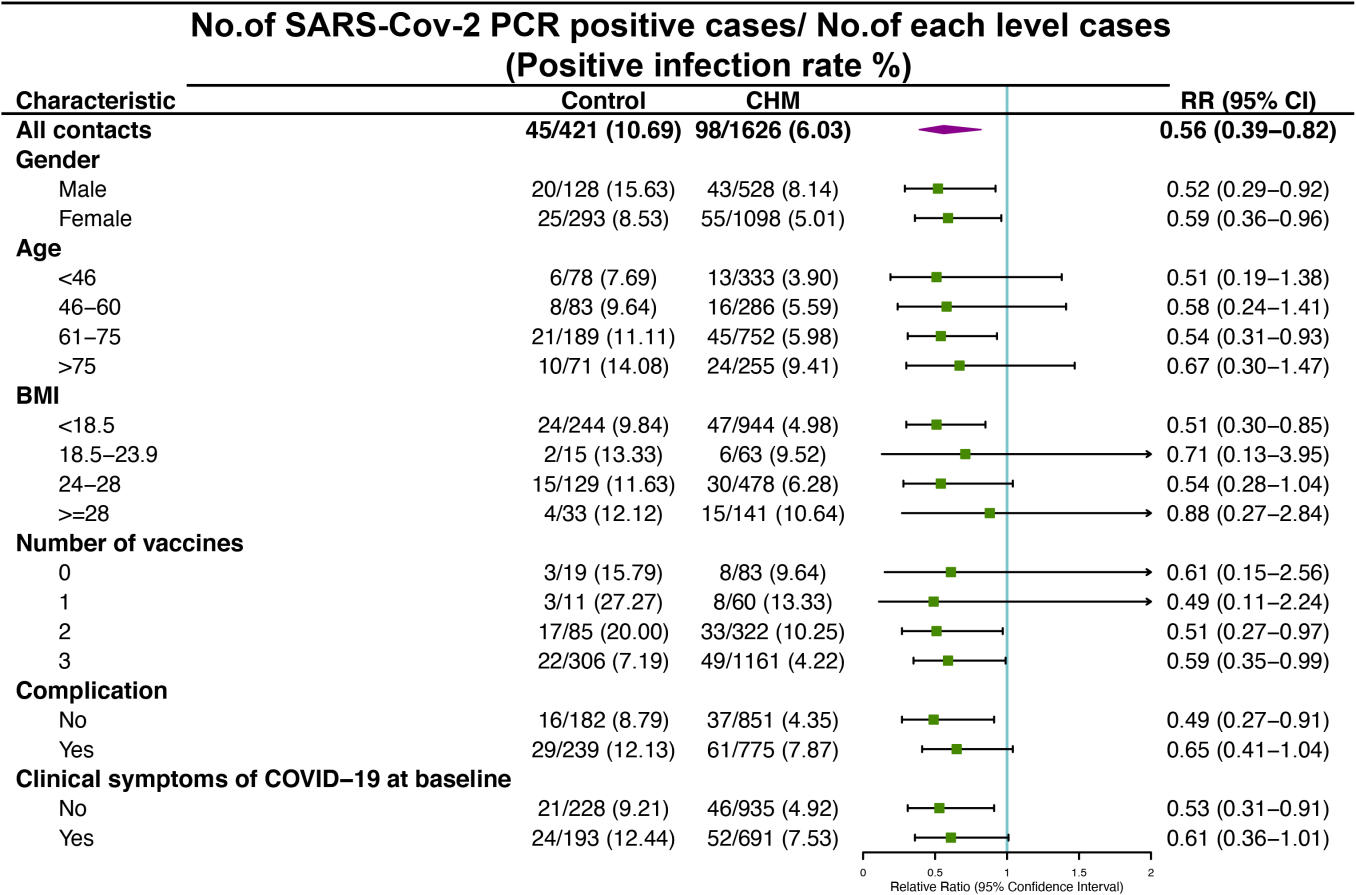
Risk of SARS-CoV-2 PCR positive among cohort A and B contacts in control and Chinese Herbal Medicine (CHM) groups after 7-day treatment period. RR, Relative Ratio; CI, Confidence Interval.

The RR of the SARS-CoV-2 PCR positive cases at the different levels of the baseline characteristics in cohort A and B are shown in **Figure 2**. During the whole 7-day treatment period, the control group had a total of 45 (10.69%) SARS-CoV-2 PCR positive cases, whereas the CHM group had 98 (6.03%) cases. The overall RR between these two groups was significant, with RR (0.56; 95% CI, 0.39-0.82). In the sub-analysis, the RR was significant in both males (0.52; 95% CI, 0.29 to 0.92) and females (0.59; 95% CI, 0.36-0.96). Among different age groups, only those aged 61 to 75 years showed a significant RR (0.54; 95% CI, 0.31-0.93). Contacts with a BMI of <18.5 in the control group had a higher risk of testing PCR positive than those in the CHM group (0.51; 95% CI, 0.30-0.85). The RR was (0.51; 95% CI, 0.27-0.97) and (0.59; 95% CI, 0.35-0.99) for the 2- and 3-dose recipients, respectively. Among the contacts, those without complications or clinical symptoms of COVID-19 at baseline exhibited an elevated RR in the control group compared to the CHM group, with RR (0.49; 95% CI, 0.27-0.91) and (0.53; 95% CI, 0.31-0.91), respectively.

Results of multivariate logistic regression model suggested that the risk of PCR positive in females was lower than in males (OR: 0.58, 95% CI: 0.38-0.89), in contacts with 3-dose injections were lower than those without vaccination (OR: 0.42, 95% CI: 0.19-0.94), and in contacts with complication were 1.78 times higher than those without complication (OR: 1.78, 95% CI: 1.13-2.78). Contacts aged 61 to 75 (OR: 3.13, 95% CI: 1.55-6.35) and over 75 (OR: 5.85, 95% CI: 2.61-13.13) were both higher risk at PCR positive compared with those aged under 46 (**Table 5**).

**Table 5:** Multivariate logistic regression the association of independent variables in CHM group with SARS-CoV-2 PCR positive.

Parameters	Coefficient(β)	S.E.(β)	OR (95%CI)	*P* value
Gender				
Male	Ref	–		
Female	-0.54	0.22	0.58 (0.38-0.89)	0.013
Age-group				
<46	Ref	–		
46-60	0.55	0.4	1.74 (0.80-3.80)	0.163
61-75	1.14	0.36	3.13 (1.55-6.35)	0.002
>75	1.77	0.41	5.85 (2.61-13.13)	<0.001
BMI-group				
18.5-24	Ref	–		
<18.5	-0.78	0.48	0.46 (0.18-1.17)	0.103
24-28	-0.72	0.5	0.49 (0.18-1.29)	0.149
>28	-0.25	0.54	0.78 (0.27-2.25)	0.645
Number of vaccines				
0	Ref	–		
1	0.56	0.56	1.76 (0.59-5.26)	0.314
2	0.36	0.44	1.44 (0.60-3.42)	0.414
3	-0.87	0.42	0.42 (0.19-0.94)	0.036
Complication				
No	Ref	–		
Yes	0.57	0.23	1.78 (1.13-2.78)	0.012
Clinical symptoms of COVID-19				
No	Ref	–		
Yes	0.45	0.28	1.56 (0.90-2.71)	0.113

CHM, Chinese Herbal Medicine; OR, odds ratio.

### Adverse events

In the CHM group, 2 contacts (0.1%) reported skin rash with itchiness, and 1 contact reported experiencing bitterness and flatulence within the 21 days period. No serious adverse events were reported.

## Discussion

In this RCT of CHM for the prevention and treatment of subjects who had close contact with confirmed COVID-19 patients, we have taken an innovative approach by categorizing the contacts into four groups according to their SARS-CoV-2 PCR test results and clinical symptoms to explore the effect of CHM among different conditions. Our findings demonstrate that CHM significantly prevented positive infection events among close contacts who were PCR-negative before the intervention, especially those without concurrent COVID-19 clinical symptoms. CHM also showed promise in accelerating the relief of clinical symptoms. Furthermore, our study found that CHM had a favorable safety profile in the prevention and treatment of COVID-19.

COVID-19 is classified as a “cold and damp epidemic virus” in TCM (28). The CHM medication used in this study comprises 10 TCM ingredients, including Jinyinhua, Lianqiao and Jiegeng, all known for their TCM effects of “soothing wind”, ventilating the lungs, and clearing away heat and toxic materials (29). Modern pharmacological study have revealed that these herbs contain chemical components like kaempferol, stigmasterol, and quercetin, which contribute to possessing antiviral, anti-inflammatory, and multiple immunomodulatory functions (30). Furthermore, Radix Isatidis polysaccharide (RIP), the crude extract of Banlangen, has been shown to reduce the mRNA expression levels of the inflammatory factors IL-6, IL-8, and IL-1β by decreasing the mRNA expression level of NF-κB (31). Given that IL-6 is one of the pivotal inflammatory factors, these TCM ingredients may mitigate the cytokine storm in COVID-19, lower the risk of disease aggravation, and improve patient outcomes. The formula applied in our study was specifically designated for COVID-19 patients and their close contacts which more than 170,000 doses of medication were distributed during the pandemic with encouraging efficacy (32).

Several previous studies have demonstrated the effectiveness of Traditional Chinese Medicine (TCM) in treating COVID-19 (19, 25). However, there is a lack of evidence regarding the effectiveness of TCM in preventing SARS-CoV-2 infection among close contacts. A few studies have reported on the effect of Lianhua Qingwen Capsules (LHQW) in close contact (33, 34). LHQW is a TCM which initially designed for treating influenza infection (25). These prior trials found that the positive infection rate of SARS-CoV-2 was significantly lower in the LHQW treatment group compared to non-LHQW group. However, these studies primarily involved close contacts from isolation settings, where SARS-CoV-2 exposure tends to be lower compared to household contacts. Consequently, the incidence of positive cases in these studies was relatively low. The CHM in this study is a TCM specifically designed for the prevention and treatment of COVID-19. Our findings revealed a household infection rate of 14.02% in the control group, slightly lower than that reported in predominately unvaccinated contacts (35). Furthermore, the CHM group exhibited a significantly lower infection rate of 8.72%, highlighting the preventive role of CHM.

It’s worth noting that previous studies consistently report a higher male representation in SARS-CoV-2 infection and more severe outcomes in males, including higher fatality rates (36, 37). Our results align with these findings, showing a higher infection rate in males during the study period. Fortunately, CHM reduced the risk of COVID-19 infection in both males and females. Besides, low BMI (<18.5 kg/m^2^) contacts appeared to benefit more from CHM in terms of prevention. A study in England suggested that individuals with low BMI had lower vaccine uptake and vaccine effectiveness (38). Therefore, CHM could be an effective option for the prevention of COVID-19 in contacts with low BMI.

In our study, the household infection rate was significantly lower in the CHM group among close contacts without complications while such significance was not observed among close contacts with complications. Previous research has highlighted that common complications such as hypertension, hyperlipidemia, diabetes, and chronic pulmonary diseases can elevate the risk of contracting COVID-19. Additionally, these complications have been shown to diminish the immune response generated by vaccines and reduce the effectiveness of clinical medications (39). Considering these findings together, tailored healthcare strategies for the prevention and management of post-infection morbidity should be specifically targeted towards close contacts who have pre-existing complications (40).

While many people look to vaccines as a key strategy for preventing disease and curbing further transmission, it’s important to acknowledge that even with vaccination, complete immunity to COVID-19 is not guaranteed. A recent review summarized that in fully vaccinated individuals, COVID-19 VE against symptomatic and asymptomatic infections was 80-90% (41). Furthermore, government statistics and academic publications have reported various specific side effects associated with vaccination (42, 43). Remarkably, our study demonstrated that CHM exhibits a higher efficacy in preventing SARS-CoV-2 infection in close contacts who have received 2- and 3- doses of vaccinations. This suggests that CHM can complement the action of vaccines and is not in conflict with them. It’s worth noting that the effectiveness of vaccines tends to wane over time, and CHM may offer a novel and valuable approach to enhance prophylactic efficacy.

There are several limitations in this study. First, due to the urgency of the epidemic and the need for timely prevention and treatment, we opted for a blank control group and did not implement blinding. Second, the treatment duration was limited to 7 days, leaving room for further investigation into whether extending the treatment period would yield increased efficacy. Third, the study focused exclusively on contacts from Hong Kong, which may limit the generalizability of the findings. Lastly, there was an absence of investigation into the biological mechanisms underlying the clinical findings in the study.

In this study, the results indicate the significant effect of CHM on decreasing the positive household infection rate of COVID-19 among close contacts. In light of their efficacy and safety profile, CHM can be considered useful for the prevention of COVID-19 upon exposure. Future double-blind, prospective, randomized controlled trials and long-term follow-up are needed to fully evaluate the efficacy of CHM in a larger contact population.

## Data availability statement

The raw data supporting the conclusions of this article will be made available by the authors, without undue reservation.

## Ethics statement

The studies involving humans were approved by the Hong Kong Baptist University Research Ethics Committee. The studies were conducted in accordance with the local legislation and institutional requirements. The participants provided their written informed consent to participate in this study.

## Author contributions

PD: Formal analysis, Writing – original draft. WL: Writing – review & editing. CL: Data curation, Writing – original draft. HL: Investigation, Writing – original draft. ZL: Data curation, Writing – original draft. CY: Methodology, Writing – original draft. CC: Visualization, Writing – original draft. TL: Data curation, Writing – original draft. ZB: Conceptualization, Project administration, Supervision, Writing – review & editing. LZ: Conceptualization, Project administration, Supervision, Writing – original draft, Writing – review & editing.
